# Editorial: Emerging Tools for Emerging Symbioses—Using Genomics Applications to Studying Endophytes

**DOI:** 10.3389/fmicb.2017.00859

**Published:** 2017-05-16

**Authors:** Mysore V. Tejesvi, Anna Maria Pirttilä, A. Carolin Frank

**Affiliations:** ^1^Ecology and Genetics, Faculty of Science, University of OuluOulu, Finland; ^2^Life and Environmental Sciences and Sierra Nevada Research Institute, School of Natural Sciences, University of California, MercedMerced, CA, USA

**Keywords:** endophytes, trancriptomics, genomics, microarrays, host–endophyte interaction

Endophytes, bacteria or fungi colonizing the interior of plants, are abundant, diverse, and play critical ecological roles across natural and agricultural ecosystems. Plants are typically colonized by hundreds of endophyte species without any noticeable disease symptoms. Endophytes have attracted the attention of researchers all over the world due to their various beneficial effects on plants. The rationale for studying endophytes using emerging tools is to better understand the beneficial bacteria and fungi associated with agricultural crops, including their origin, maintenance, and nutritional requirements could help the development of sustainable agricultural systems and the exploitation of various plant–symbiont natural products. Recent advances in DNA sequencing technology and computation now allow more complete descriptions of the endophyte community composition without the biases imposed by culturing. Complementing taxonomic data, community functional traits such as substrate utilization, can be characterized at high throughput using Phenotype MicroArrays (Blumenstein et al.). Comparing whole genomes of endophytes and other symbiotic bacteria with those of pathogens, the factors underlying symbiosis can be identified. Furthermore, analysis of the transcriptomes of both host and microbes can unveil the molecular underpinnings of the interaction.

The studies collected in this ebook advance our knowledge of endophytes and their impact on plants, highlighting the importance of both bacterial and fungal microbiomes on plant health and growth. For example, the largest plants on Earth, *Sequoia sempervirens* and *Sequoiadendron giganteum* host variable communities of bacteria in their foliage, including *Bacillus, Burkholderia*, and *Actinomycetes* species that could protect the giant trees against biotic stress was studied using 454 sequencing technology and data was analyzed using Quantitative insights into Microbial Ecology (QIIME) package (Carrell and Frank). It is already known that many endophytes induce resistance and protect the host against diseases. An endophyte of olive, *Pseudomonas fluorescens* PICF7, was found by Gómez-Lama Cabanás et al. antagonistic toward *Verticillium*, triggering a broad range of defense responses in root tissues and was studied using Suppression Subtractive Hybridization” (SSH) technology and quantitative real-time PCR (qRT-PCR). Results indicated that the endophyte triggered defense responses not only locally in roots, but also in other tissues, such as stems and leaves, indicating a systemic response. Host responses by dark septate endophytes (DSEs) were elucidated in the model plants *Arabidopsis thaliana* and *Allium porrum*, showing to be dependent on the plant species and ecotypes. An important finding was that the shifts in the environmental conditions directed the host responses along the mutualism–parasitism continuum, which confirms earlier understanding of the thin line between an endophyte and a pathogen (Mandyam and Jumpponen).

Interaction and co-existence of endophytes and pathogens often depends on the full plant microbiome and virulence factors. One of the hot topics in our book is the comparative genomics of pathogens and endophytes, which is aimed to get a better grasp of these complex interactions and in turn to lead to a better understanding of plant health. Major differences between genomic virulence factors of endophytes and non-endophytes were analyzed by Lòpez-Fernàndez et al. whereas Sheibani-Tezerji et al. compared the genomes of three strains of *Pantoea ananatis*, isolated from healthy maize seeds, to identify functional genes and the genetic drivers of niche adaption.

Many endophytes have other beneficial effects on the plant host besides protection against diseases, such as growth promotion. Some endophytes can induce development of roots or shoots of the host plant and the composition of the endophytic community may change along with development. For example, *Agrobacterium* and *Erwinia* dominated the seedling stage and members of *Sphingomonas* and *Methylobacterium* were mainly found in the mature leaves of Stevia, positively correlating with *Steviol glycosides* accumulation and was studied using V4 and V6 region of 16S rRNA gene using 454 GS-FLX platform (Yu et al.). Whereas the endophytes typically studied and sequenced are bacteria or fungi, the specific group of endophytic yeasts has largely gone unstudied. The pink-pigmented yeast strain *Rhodotorula graminis* WP1, isolated from *Populus trichocarpa*, was the *first* endophytic yeast genome to be sequenced using the Sanger whole genome shotgun approach (Firrincieli et al.). In comparison with the mycorrhizal fungus *Laccaria bicolor, R. graminis* WP1 potentially uses a different signaling pathway to communicate with the host, even though both of these fungi are Basidiomycete symbionts of poplar. Another example is the study by Tadra-Sfeir et al. where RNA-Seq was used to identify the essential genes required for establishment and interaction of endophytes in the transcriptome of the endophytic diazotroph *Herbaspirillum seropedicae*.

The endophytic microbiomes have growing significance in applications such as agriculture and forestry, and may play important but yet unrecognized roles in climate change. In the banana microbiome studied using Illumina MiSeq Sequencing, differences in indicator species associated with legume-based agroforestry (Köberl et al.) suggest that there is a flow between the microbiomes of neighboring plants. This brings the cropping systems to a completely new era as not only nitrogen fixation, but other beneficial traits, such as biocontrol, can be acquired from surrounding plant communities. A serious implication of climate change on methane emissions by rice microbiomes was studied using 454 GS FLX sequencing platform by Okubo et al. The relative abundance of methane-oxidizing *Methylocystaceae* decreased in the rice plants with increased CO_2_ levels. As an alarming result, the elevated CO_2_ affected the carbon cycle in the rice paddy fields by suppression of methane oxidation and through increased methanogenesis.

This e-book represents an outstanding collection of endophytic research done by various modern molecular biology tools, including next generation sequencing, genome sequencing, and transcriptomics. The book advances our knowledge and understanding on important elements of this symbiosis and provides food for thought for future research. We are grateful to all the authors who have responded to the call and for reviewers for their valuable support.

## Author contributions

Editorial was written by MT and subsequent revision, discussion, and modifications were done by MT, AMP, and ACF.

### Conflict of interest statement

The authors declare that the research was conducted in the absence of any commercial or financial relationships that could be construed as a potential conflict of interest.

